# Investigating the Association Between Fusobacterium nucleatum and Oral Squamous Cell Carcinoma: A Pilot Case-Control Study on Tissue Samples

**DOI:** 10.7759/cureus.47238

**Published:** 2023-10-17

**Authors:** Sriram Kaliamoorthy, Sugantha Priya Sayeeram, Shanmugapriya SundarRaj, Jeyakumar Balakrishnan, Mahendirakumar Nagarajan, Agila Samidorai

**Affiliations:** 1 Dentistry, Vinayaka Mission's Medical College and Hospital, Vinayaka Mission’s Research Foundation (DU), Karaikal, IND; 2 Dental Surgery, Thanjavur Medical College, The Tamilnadu Dr. M.G.R Medical University, Thanjavur, IND; 3 Central Research Laboratory, Vinayaka Mission’s Medical College and Hospital, Vinayaka Mission’s Research Foundation (DU), Karaikal, IND; 4 Prosthodontics and Crown & Bridge, Cuddalore Government Dental College, The Tamilnadu Dr. M.G.R Medical University, Chidambaram, IND; 5 Periodontics, Chettinad Dental College and Research Institute, The Tamilnadu Dr. M.G.R Medical University, Chennai, IND

**Keywords:** tumor microenvironment, polymerase chain reaction, oral squamous cell carcinoma, cancer, fusobacterium nucleatum, human microbiome

## Abstract

Background

*Fusobacterium nucleatum *(*F. nucleatum*)* *has been increasingly linked to oral squamous cell carcinoma (OSCC), prompting this study to explore its presence using polymerase chain reaction (PCR) and evaluate its clinical significance.

Methods

In this pilot case-control study, 12 OSCC tissue samples and 12 non-cancerous oral mucosal tissue samples were analyzed. Total RNA extraction and complementary DNA (cDNA) synthesis were performed using Trizol-based methods, followed by PCR amplification and gel electrophoresis. The clinical characteristics of participants and PCR results were recorded.

Results

Among the OSCC tissue samples, three out of 12 tested positive for *F. nucleatum,* while none of the control samples showed its presence. The detection rate of *F. nucleatum* in OSCC was 25%. Gel analysis confirmed specific amplicon amplification, and ImageJ software enabled copy number quantification.

Discussion

Our findings support previous research indicating a potential association between *F. nucleatum* and OSCC. Understanding the etiological significance of *F. nucleatum* in OSCC has clinical implications, including early detection, risk stratification, and prognostication. However, the limited sample size and the need for further research to elucidate underlying mechanisms are acknowledged.

Conclusion

This pilot study provides initial evidence of *F. nucleatum’s* presence in a subset of OSCC samples, supporting its potential association with oral cancer. Detecting *F. nucleatum *in OSCC tissues holds promise for future research and clinical applications as a diagnostic and prognostic biomarker. Understanding its role in oral carcinogenesis will facilitate the development of targeted therapeutic strategies. Larger studies are warranted to validate these findings and investigate the precise mechanisms involved.

## Introduction

The human microbiome, particularly *Fusobacterium nucleatum* (*F. nucleatum*), is increasingly recognized as relevant to cancer development. *F. nucleatum*, commonly found in the oral cavity, has been associated with various cancers, including oral squamous cell carcinoma (OSCC), where it influences the tumor microenvironment and promotes tumorigenesis [1-3]. OSCC, originating from oral cavity epithelial cells, has established risk factors such as tobacco and alcohol. Recent studies suggest a potential connection between the oral microbiome and OSCC development [4-6]. To investigate *F. nucleatum*’s presence and abundance in OSCC tissue samples, polymerase chain reaction (PCR) will be used for DNA amplification and detection. By targeting specific genetic markers in the *F. nucleatum* genome, PCR-based methods aim to quantify its presence in diverse OSCC specimens.

This study aims to establish an association between *F. nucleatum* and OSCC, expanding our understanding of the oral microbiome’s role in cancer development. Detecting *F. nucleatum* in OSCC tissue samples has significant clinical implications, potentially serving as a diagnostic biomarker for early detection and risk stratification of OSCC [7,8]. Additionally, identifying *F. nucleatum* in OSCC may provide prognostic value, guide treatment decisions, and predict patient outcomes. Understanding how *F. nucleatum* influences OSCC development may lead to novel therapeutic targets and improved patient care.

This research investigates the presence, role, and clinical significance of *F. nucleatum* in OSCC, contributing to our understanding of the oral microbiome’s involvement in cancer and potentially enhancing patient management.

## Materials and methods

Study design and sample collection

This pilot case-control study aimed to investigate differential gene expression between OSCC samples and non-cancerous normal oral mucosal tissue samples. A total of 24 tissue samples were included in this study, comprising 12 OSCC samples and 12 non-cancerous normal oral mucosal tissue samples. Purposive sampling was employed to select these samples, ensuring a representative cross-section of both OSCC and normal tissues. Prior to sample collection, informed consent was diligently obtained from all participants.

Tissue preparation and storage

Tissue samples were meticulously collected, following standard sterile procedures. Each sample was washed thoroughly with sterile 1X phosphate-buffered saline (PBS) to remove any contaminants or debris. Subsequently, the samples were carefully preserved in Trizol reagent (Thermo Fisher Scientific, Waltham, MA) and immediately stored at −20°C to maintain RNA integrity until further analysis.

RNA extraction and complementary DNA (cDNA) synthesis

Total RNA extraction from the tissue samples was performed using the highly regarded Trizol-based techniques, as recommended by Thermo Fisher Scientific. This method was chosen for its reliability and effectiveness in isolating high-quality RNA. Subsequently, the concentration and quality of the extracted RNA were meticulously assessed using a NanoDrop spectrophotometer, ensuring that only high-quality RNA was utilized in subsequent steps.

For cDNA synthesis, 500 ng of the purified RNA was reverse transcribed using RevertAid reverse transcriptase from Thermo Scientific, Waltham, MA. This step was carried out with precision to ensure the generation of cDNA representative of the original RNA, allowing for downstream gene expression analysis.

PCR amplification

PCR amplification was performed with great care using a red dye master mix from Ampliqon, Odense, Denmark. This step aimed to selectively amplify the target gene sequences from the cDNA, providing the necessary material for subsequent analysis. PCR conditions were optimized to ensure specific and reproducible amplification of the target genes.

Analysis of PCR products

The PCR products were analyzed using electrophoresis on agarose gels stained with ethidium bromide. The gel electrophoresis setup was carefully prepared to maintain the integrity of the samples. Following electrophoresis, gel images were captured and analyzed using ImageJ software, a widely recognized image analysis tool.

The unknown concentration of DNA was determined using ImageJ’s pixel density analysis based on a known DNA ladder. To calculate the concentration in each sample, we employed a formula that considers the average weight of a DNA base pair (bp) to be 650 Da. This implies that the molecular weight of any double-stranded DNA template can be estimated by multiplying its length (in bp) by 650. The reciprocal of this molecular weight gives us the number of moles of the template present in one gram of material. Using Avogadro’s number, which is 6.022 × 10^23^ molecules per mole, we can then calculate the number of template molecules per gram. To estimate the number of molecules or copies of the template in the sample, we multiply this result by 1 × 10^9^ to convert to nanograms (ng) and then by the amount of template in ng. In essence, this calculation involves inputting the template’s ng amount and its bp length into the formula below to determine the number of template copies: number of copies = (amount in ng × 6.022 × 10^23^)/(length in bp × 1 × 10^9^ × 650). By utilizing this formula, you can easily ascertain the number of template copies in your sample.

Ethical clearance

Ethical clearance for this study was obtained from the institutional review board, following established guidelines and protocols for human subject research. The study adhered to all ethical principles and regulations governing research involving human participants, ensuring the protection of their rights, privacy, and well-being.

## Results

This pilot study included a cohort of 12 patients diagnosed with primary OSCC as cases, alongside an equal number of healthy tissue samples serving as controls. The patient demographics were characterized by a gender distribution of seven males and five females among the OSCC cases. Males had a mean age of 57, while females had a mean age of 67. In the control group, there was a balanced representation of both males and females, with males averaging 36 years and females averaging 42 years in age.

Within the cohort of OSCC patients, clinical staging was observed as follows: stage 3 (n = 7), stage 2 (n = 3), and stage 4 (n = 2). Predominantly, OSCC occurred most frequently within the right alveobuccal complex (n = 8), followed closely by the left alveobuccal complex (n = 4).

The study employed PCR experiments to ascertain the presence of *F. nucleatum* in the tissue samples. Out of the 12 OSCC subjects, *F. nucleatum* was identified in three cases, signifying a detection rate of 25%. This finding posits a potential correlation between *F. nucleatum* and OSCC.

**Figure 1:**
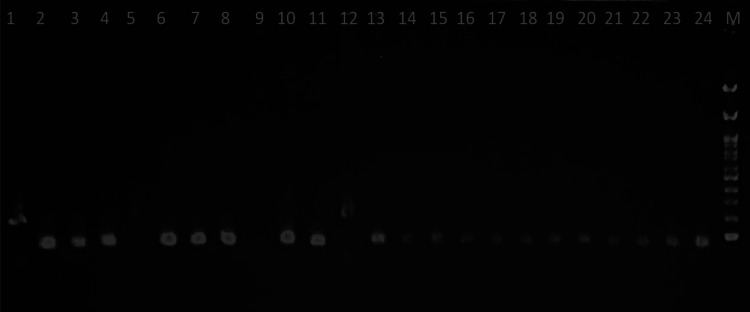
PCR detection of F. nucleatum in oral squamous carcinoma samples (lanes 1-12) and control samples (lanes 13-24) Positive amplification was detected in samples 1, 5, and 12 (lanes 1, 5, and 12) PCR, polymerase chain reaction

To validate the specificity of amplicon amplification, gel analysis was meticulously conducted. The gel electrophoresis results prominently displayed distinct bands that corresponded precisely to the target *F. nucleatum* genetic markers (Figure 1). This unequivocally confirms the successful amplification of *F. nucleatum* DNA within the positive cases (Figure 1).

**Figure 2:**
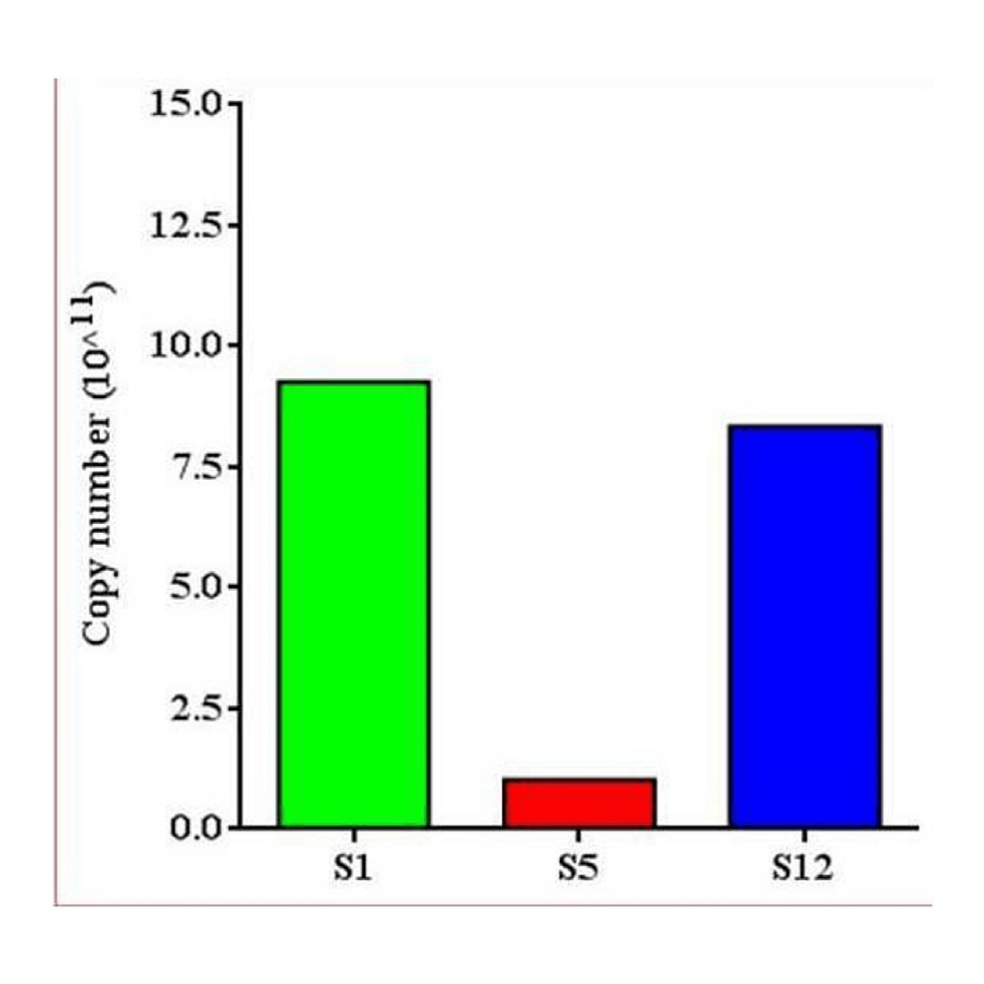
Quantitation of F. nucleatum DNA in oral cancer tissue samples through PCR PCR, polymerase chain reaction

In order to precisely quantify the abundance of *F. nucleatum* within the positive cases, we harnessed the capabilities of ImageJ software for image analysis (Figure 2). This analysis method allowed for the accurate quantification of *F. nucleatum* DNA copy numbers within the OSCC tissue samples.

These results furnish us with initial evidence that supports the presence of *F. nucleatum* within a subset of OSCC cases. A detection rate of 25% implies a conceivable association between *F. nucleatum* and OSCC, thereby advocating for further exploration through large-scale studies. The affirmative confirmation of specific amplicon amplification, coupled with the accurate quantification of *F. nucleatum* DNA copy numbers, substantially fortifies the credibility of these findings.

## Discussion

This pilot study sought to shed light on the potential association between *F. nucleatum* and OSCC through the application of PCR analysis. Our investigation revealed that out of the 12 OSCC samples analyzed, three tested positive for the presence of *F. nucleatum*, while none of the control samples exhibited this bacterium. These initial findings are in line with existing research, underscoring the potential link between *F. nucleatum* and oral cancer [9-11].

Our results align with the observations made by Nagy et al. (1998), who reported a greater abundance of aerobic and anaerobic colony-forming units, including *F. nucleatum*, in biofilms associated with OSCC as compared to those from healthy mucosal tissues [9]. Furthermore, studies conducted by Yang et al. (2018) and Han et al. (2019) have consistently identified *F. nucleatum* within OSCC tissues, providing additional support for its potential role in cancer initiation and progression [10,11].

These findings collectively underscore the plausibility of *F. nucleatum*’s involvement in OSCC, echoing the conclusions drawn in the comprehensive review by McIlvanna et al. (2021), which presented compelling evidence supporting the association between *F. nucleatum* colonization and the heightened risk of OSCC [12].

However, it is essential to acknowledge the limitations of our pilot study. The foremost limitation lies in the small sample size utilized, which may not fully represent the broader population. Therefore, our findings should be interpreted cautiously and considered preliminary. To establish the robustness and generalizability of these results, larger-scale studies encompassing a more extensive and diverse cohort are warranted.

Furthermore, while our study has provided a glimpse into the potential link between *F. nucleatum* and OSCC, the precise mechanisms underpinning *F. nucleatum*’s contribution to oral carcinogenesis remain enigmatic. The molecular and biological pathways through which *F. nucleatum* may influence cancer development and progression require further exploration.

## Conclusions

Our pilot case-control study offers preliminary evidence suggesting a potential association between *F. nucleatum* and OSCC. These findings resonate with prior research and highlight the need for more extensive investigations to confirm and extend our understanding of this association. Moreover, unraveling the intricate mechanisms governing *F. nucleatum*’s role in oral carcinogenesis is essential for developing targeted interventions and therapies that may ultimately benefit individuals at risk of developing OSSC.
